# Clinical applications of multiparametric MRI within the prostate cancer diagnostic pathway^☆^

**DOI:** 10.1016/j.urolonc.2012.02.004

**Published:** 2013-04

**Authors:** Louise Dickinson, Hashim U. Ahmed, Clare Allen, Jelle O. Barentsz, Brendan Carey, Jurgen J. Futterer, Stijn W. Heijmink, Peter Hoskin, Alex P. Kirkham, Anwar R. Padhani, Raj Persad, Philippe Puech, Shonit Punwani, Aslam Sohaib, Bertrand Tombal, Jan van der Meulen, Arnauld Villers, Mark Emberton, (PREDICT Consensus Panel)

**Affiliations:** a Division of Surgery and Interventional Sciences, University College London, Gower Street, London, UK; b Department of Urology, University College London Hospitals NHS Foundation Trust, 235 Euston Road, London, UK; c Clinical Effectiveness Unit, Royal College of Surgeons of England, 35-43 Lincoln's Inn Fields, London, UK; d Department of Radiology, University College London Hospitals NHS Foundation Trust, 235 Euston Road, London, UK; e Department of Radiology, Radboud University Nijmegen, Medical Centre, 6500 HB, Nijmegen, the Netherlands; f Department of Radiology, Leeds Teaching Hospitals, NHS Trust, Beckett Street, Leeds, UK; g Cancer Centre, Mount Vernon Cancer Centre, Rickmansworth Road, Northwood, Middlesex, UK; h Paul Strickland Scanner Centre, Mount Vernon Cancer Centre, Rickmansworth Road, Northwood, Middlesex, UK; i Department of Urology, University Hospitals Bristol NHS Foundation Trust, Marlborough Street, Bristol, UK; j Department of Radiology, CHU Lille Univ Lille Nord de France, F-59000 Lille, France; k Department of Radiology, Royal Marsden NHS Foundation Trust, Fulham Road, London, UK; l Department of Urology, Cliniques Universitaires Saint-Luc, Avenue Hippocrate 10, 1200 Brussels, Belgium; m Health Services Research Unit, London School of Hygiene and Tropical Medicine, UK; n Department of Urology, CHU Lille Univ Lille Nord de France, F-59000 Lille, France

Interest in integrating MRI into the prostate (CaP) cancer diagnostic pathway seems to be gaining ground [1]. Multiparametric (mp)MRI combines diffusion-weighted, dynamic contrast enhanced sequences or MR spectroscopy with conventional T2-weighted sequences. This has resulted in accuracy rates for the detection of clinically important CaP that compare favorably with established tests, such as X-ray mammography, for the detection of breast cancer [1,2]. However, its exact clinical utility remains the subject of legitimate professional disagreement. In this commentary, we attempt to highlight the areas in which mpMRI may have a role in improving the diagnosis and management of CaP.

## Multiparametric MRI in response to a negative first biopsy

The main reason why the current diagnostic pathway for CaP remains suboptimal is that the current standard, transrectal ultrasound (TRUS) guided biopsy, is conducted ‘blind’ to the cancer location within the prostate. Men who enter the current CaP diagnostic pathway on the basis of an elevated serum prostatic specific antigen (PSA) level have a 1 in 4 chance of testing positive on TRUS guided biopsy. More men, therefore, are subjected to TRUS guided biopsies than is probably necessary. This renders CaP diagnosis a health state that is largely determined by chance. In fact, it is slightly worse. Cancers, in the anterior prostate, apex, and midline are either undersampled or never sampled, resulting in clinically significant cancers going undetected [3,4].

Because of this diagnostic strategy, many men are falsely reassured that they are free of clinically significant cancer when they are not. By using mpMRI to assess the risk status of men with a previous negative biopsy, biopsies can be targeted to visible MR lesions [5]. When this strategy has been used, almost two-thirds (59%) of men with 2 or more previous negative TRUS biopsies have been diagnosed with cancer [6].

## Multiparametric MRI as a triage test for men at risk

The recent attribution of a grade D by the U.S. Preventive Services Task Force against PSA screening (in other words of ‘moderate or high certainty that the service has no net benefit or that the harm outweighs the benefits’) would have us believe that PSA is not fit for the purpose [7]. However, it may be that the application of the verification test that follows PSA requires scrutiny rather than rejection of PSA screening altogether. In introducing imaging earlier in the diagnostic pathway, before TRUS guided biopsy, in those men with an elevated serum PSA or other risk factors, urologists would conform to the practice adopted by clinicians treating other solid organ cancers [8].

For example, screening X-ray mammograms and breast MRI scans performed for ‘at-risk' women are scored according to the Breast Imaging-Reporting and Data System (BI-RADS) [9,10]. Scored between 0 and 5 according to the likelihood that cancer is present, this reporting system was developed by a consensus group endorsed by the American College of Radiology to standardize and quality control the reporting of breast imaging. Those with an equivocal score of 3 would be recommended to have further follow-up within a few months, while a score of at 4 or 5 denotes suspicion for malignancy warranting biopsy directed towards the area of suspicion. It would appear feasible for the uro-oncology community to adopt the same approach, with fewer and better image-targeted biopsies performed in men with a positive mpMRI [11,12]. In those with an equivocal mpMRI, PSA surveillance or standard TRUS guided biopsy would be recommended, while scores of 2 and below may warrant no further follow-up, depending on individual patient and clinician preference.

One of the key attributes of modern imaging platforms in cancer diagnosis is that the result correlates closely with tumor burden. Multiparametric MRI has reduced sensitivity for low grade, low-volume disease and, therefore, may systematically overlook clinically insignificant disease. If this proves to be the case, its application as a triage test before prostate biopsy might significantly address the problem of overdiagnosis that is associated with the current diagnostic pathway. Evidence is starting to accumulate that mpMRI may be sensitive to tumor grade as well as tumor volume [13–17], resulting in a high negative predictive value for ruling out clinically significant disease [18]. This strategy would reduce the incidence of treatment-related harm encountered by overdiagnosis of low-risk disease—the main reason that the U.S.A. task force recommended against the use of PSA screening.

## Cancer localization on multiparametric MRI

Approximately one-third of patients undergoing active surveillance have upgrading of disease on serial TRUS guided biopsies [19,20]. In some men, this change is related to true disease progression. However, in others it reflects the inadequacy of a sampling biopsy technique to provide consistent serial information on a patient's pathologic status. As an alternative, if mpMRI can accurately detect, localize, and characterize tumors, we have the opportunity to monitor a true and visible change in a lesion over time. There is evidence that mpMRI can act as an accurate monitoring tool for CaP progression in those men undergoing active surveillance [21].

Accurate cancer localization may also aid the optimal balance between oncologic outcome and genitourinary and rectal side effects in nerve-sparing radical prostatectomy [22] and dose escalation to the index lesion in radiotherapy [23]. Novel experimental strategies to selectively treat the cancer alone also rely on imaging. For instance, real-time mpMRI has been used to direct focal treatments with laser in Phase I/II trials [24].

## Future studies

Despite agreement across the uro-radiology community that mpMRI has a potential future in the standard diagnostic pathway for CaP, differences in current conduct, interpretation, and reporting of mpMRI renders reliable comparison of research studies difficult [25,26]. This is currently an impediment to widespread and effective adoption of this technique. However, there are signs of change. Consensus now exists on the minimal and optimal imaging standards for mpMRI [25], and clinical practice guidelines have recently been produced [27].

Once agreed, standardized mpMRI protocols and reporting schemes will require validation within prospective studies that evaluate the ability of mpMRI to detect, localize, and characterize CaP against an appropriate reference standard. Such a trial is due to start imminently (http://clinicaltrials.gov NCT01292291).

Targeting of biopsies to lesions identified on mpMRI is already in clinical use in a number of centers, but the techniques adopted are varied. Some groups use ‘cognitive’ registration of the results of the mpMRI to target biopsies on TRUS or transperineal prostate biopsies [28]. Prostate distortion and poor interpretation of imaging are potential limitations. Other groups are evaluating ‘in-bore' targeting of lesions within the MR scanner, and have demonstrated improved cancer detection rates [5,6], and risk stratification of disease [29], compared with standard TRUS biopsies. However, this technique carries a high burden on imaging resources and time, and usually requires a general anesthetic. An alternative approach is to register MR images onto an ultrasound platform, to allow real-time targeting of lesions in the theater or out-patient clinical setting [11]. The key to accuracy of this technique is in the development of ‘nonrigid’ registration that allows for movement and distortion between prostate images. Such techniques are under evaluation [30,31].

Long-term cost-of-care evaluation of integrating mpMRI into the diagnostic pathway is difficult to model, as many assumptions need to be incorporated. While transfer of costs to other sectors of the health care community is guaranteed, the impact on costs-of-care is more difficult to assume. If a new test, such as mpMRI, could deliver fewer biopsies, better biopsies, better risk stratification, more appropriate treatment allocation, fewer diagnoses, and fewer men treated overall, we might have a test that could impart significant cost savings over decades. Moreover, the capital expenditure has already been made in many parts of the world as appropriate MR scanners are already in place. The challenge will be to use the existing MRI facilities more effectively and to refine the optimal sequences for prostate so that scan time is minimized. Development of a healthcare economic model to evaluate cost-effectiveness will be required before further conclusions can be drawn about the potential long-term cost-savings of this strategy.

The integration of mpMRI within the diagnostic pathway for CaP has been embraced for some time by a number of centers in the world. There is likely to be further dissemination once practice guidelines start making recommendations based on good evidence—something that we shall see in the next couple of years. Clinicians involved in CaP diagnosis and staging should take this opportunity to begin to formulate good working relationships with uro-radiologists. This is going to be the key partnership of the future that will result in better care for men at risk.
